# Effects of Protein Restriction and Subsequent Realimentation on Body Composition, Gut Microbiota and Metabolite Profiles in Weaned Piglets

**DOI:** 10.3390/ani11030686

**Published:** 2021-03-04

**Authors:** Lei Hou, Li Wang, Yueqin Qiu, YunXia Xiong, Hao Xiao, Hongbo Yi, Xiaolu Wen, Zeling Lin, Zhikang Wang, Xuefen Yang, Zongyong Jiang

**Affiliations:** 1 Institute of Animal Nutrition, Northeast Agricultural University, Harbin 150030, China; rhoulei@126.com; 2 Institute of Animal Science, Guangdong Academy of Agricultural Sciences, State Key Laboratory of Livestock and Poultry Breeding, Ministry of Agriculture Key Laboratory of Animal Nutrition and Feed Science in South China, Guangdong Provincial Key Laboratory of Animal Breeding and Nutrition, Maoming Branch, Guangdong Laboratory for Lingnan Modern Agriculture, Guangzhou 510640, China; qiuyueqin87@126.com (Y.Q.); xiangtang.2000@163.com (Y.X.); xiaohao@gdaas.cn (H.X.); yihongbo@gdaas.cn (H.Y.); wenxiaolu@gdaas.cn (X.W.); dering@126.com (Z.L.); wongzhikang@163.com (Z.W.);yangxuefen@gdaas.cn (X.Y.)

**Keywords:** body composition, metabolite, microbiota, protein restriction, realimentation, piglet

## Abstract

**Simple Summary:**

Protein restriction strategies are often used in weaned piglets to reduce the incidence of intestinal disorders that are sensitive to dietary protein supply, but may lead to a decline in production performance. Subsequent protein realimentation can alleviate the detrimental effects of reduced dietary protein on growth. However, the effects of protein realimentation on the body composition, gut microbiota and metabolite profiles of piglets are poorly understood. The present study, combining comparative slaughter methods, microbiome and metabolome analyses, demonstrated that protein restriction and subsequent realimentation lead to compensatory growth and compensatory protein deposition in piglets, and contribute to animal intestinal health by altering the gut microbiota and metabolite profiles.

**Abstract:**

The objective of this study was to evaluate the effects of protein restriction and subsequent protein realimentation on the body composition, gut microbiota and metabolite profiles of piglets. Fifty weaned piglets were randomly assigned to two treatments: a normal protein (NP) group (20% crude protein (CP)) or a low protein (LP) group (16% CP) with five animals per pen and five pens per group. Treatment diets were fed for 14 d during the protein restriction phase, and then all pigs were fed the same nursery diets with a normal CP level (19% CP) during the protein realimentation phase until they reached an average target body weight (BW) of 25 ± 0.15 kg. At day 14 and the end of the experiment, one piglet close to the average BW of each pen was slaughtered to determine body composition, microbial composition and microbial metabolites. Results showed that there was no difference (*p* > 0.05) in the experimental days to reach target BW between the LP and NP groups. The average daily gain (ADG) and gain:feed ratio (G:F) during the protein restriction phase as well as BW at day 14, were significantly decreased (*p* < 0.05) in the LP group compared with the NP group. However, there were no significant differences (*p* > 0.05) during the protein realimentation phase and the overall experiment. Similarly, piglets in the LP group showed a significantly decreased body protein content (*p* < 0.05) at day 14, but not (*p* > 0.05) at the end of the experiment. The relative abundance of *Parabacteroides*, *Butyricicoccus*, *Olsenella*, *Succinivibrio* and *Pseudoramibacter* were significantly increased (*p* < 0.05), while the relative abundance of *Alloprevotella* and *Faecalicoccus* were significantly decreased (*p* < 0.05) in the LP group at day 14. At the end of the experiment, the piglets in the LP group showed a higher (*p* < 0.05) colonic relative abundances of *Parabacteroides*, unidentified *Christensenellaceae* and *Caproiciproducens*, and a lower (*p* < 0.05) relative abundance of unidentified *Prevotellaceae*, *Haemophilus*, *Marvinbryantia*, *Faecalibaculum*, *Neisseria* and *Dubosiella* than those in the NP group. Metabolomics analyses indicated that tryptophan metabolism and vitamin metabolism were enriched in the LP group at day 14, and glycerophospholipid metabolism and fatty acid esters of hydroxy fatty acid metabolism were enriched at the end of the experiment. Moreover, Spearman’s correlation analysis demonstrated that the microbial composition was highly correlated with changes in colonic metabolites. Collectively, these results indicated that protein restriction and subsequent realimentation lead to compensatory growth and compensatory protein deposition in piglets and contribute to animal intestinal health by altering the gut microbiota and its metabolites.

## 1. Introduction

Weaning is a critical period in the life of a pig because it is often associated with a high incidence of intestinal disorders, such as post-weaning diarrhea, resulting in growth retardation or even death [1]. It is generally assumed that intestinal disorders of newly weaned piglets are sensitive to dietary crude protein (CP) intake, and the risk of intestinal disorders may be decreased by early protein restriction [2,3]. However, a deficient dietary CP supply can result in a reduction in piglet performance [4,5]. It has also been reported that animals can grow rapidly by being supplied with sufficient nutrients in the subsequent stage after previous malnutrition or artificial restriction, which is known as the concept of compensatory growth [6,7]. However, these studies were usually based on production traits such as body weight (BW) to evaluate treatment effects without considering carcass traits such as body composition. Protein nutrition is of paramount importance for muscle growth in pigs, especially in piglets, due to their tremendous capacity for rapid growth and body protein deposition [8]. Numerous studies have consistently reported that growing pigs and fattening pigs fed with low-protein diets had increased back-fat thickness and a reduced loin muscle area [9,10,11]. However, it is unclear whether protein restriction and subsequent protein realimentation have an effect on the body composition of piglets.

The gut microbiota plays an important role in regulating the digestion and absorption of nutrients and maintaining the homeostasis of the intestinal environment [12]. It has been reported that nutrient modulation alters host physiology and metabolism through interaction with the gut microbiota and numerous bioactive bacterial metabolites [13,14]. However, the effect of protein restriction and subsequent protein realimentation on gut microbiota and bacterial metabolites in weaned piglets remains unclear.

Therefore, the current study investigated the effects of protein restriction and subsequent protein realimentation on body composition, gut microbiota and microbial metabolites in piglets by using a comparative slaughter method, and a 16S rDNA sequencing strategy and combined this with the liquid chromatograph-mass spectrometry (LC-MS) technique.

## 2. Materials and Methods

The experimental protocols and procedures performed in this study were approved by the Animal Care and Use Committee at Guangdong Academy of Agricultural Sciences (authorization number GAASIAS-2016-017).

### 2.1. Animals and Experimental Design

Fifty weaned piglets (21 days of age; 6.39 ± 0.02 kg BW; Duroc × Landrace × Yorkshire) were randomly assigned to 2 treatments: a normal protein group (NP, 20% CP) or a low protein group (LP, 16% CP) with 5 animals per pen and 5 pens (3 pens with 3 barrows and 2 gilts, 2 pens with 2 barrows and 3 gilts) per group. Crystalline amino acids (AA) were supplemented to ensure that the amount of standardized ileal digestible (SID) AA to meet or exceed the National Research Council (NRC) recommendations [15]. Treatment diets were formulated to contain similar net energy (NE) contents and were fed to piglets for 14 d. After the restriction phase, all piglets were fed the same nursery diets with a normal CP level of 19% during the realimentation phase until they reached an average target BW of 25 ± 0.15 kg. Ingredient composition and nutrient composition of the diets are presented in Table 1.

Piglets were housed in a nursery facility (2.20 × 1.50 m^2^), which had a hard plastic fully slatted floor, a multi-hole stainless feeder, and a single bowl drinker. Pigs had free access to feed and water throughout the experiment period.

### 2.2. Sample Collection

Piglets were individually weighed at the beginning of the experiment and at the end of each period to calculate average daily gain (ADG), average daily feed intake (ADFI) and gain:feed ratio (G:F), and were checked daily for the occurrence of diarrhea during the experimental period. At day 14 of the experiment and the day when pigs had an average target BW of 25 ± 0.15 kg, after collecting blood samples from the anterior vena cava, one piglet close to the average BW of each pen (3 barrows and 2 gilts per group) was anesthetized with sodium pentobarbital and was slaughtered after an overnight fast. Serum from each sample was harvested by centrifugation at 3000× *g* for 15 min at 4 °C and then stored at −80 °C for further analysis. Slaughter procedures were performed according to the previous study [16]. The digesta from the colon was immediately collected and frozen in liquid nitrogen, and then stored at −80 °C for bacterial DNA isolation and metabolite analysis. In order to obtain the empty body of a piglet for body composition measurement, the remaining digesta from the stomach and intestines were washed, and the contents from the bladder and gallbladder were removed, then were then patted dry. All carcasses were stored at −20 °C until further processing. The frozen carcass was cut into small blocks in a double-shaft crusher (L-SP380, LiWill Co. Ltd., Zhengzhou, China), then ground in a grinder with an 18-mm die (SG-130, Yusheng Co., Xingtai, China) and finally ground using an ultrafine grinding mill (GN-130, Yusheng Co., Xingtai, China). The ground carcass was mixed in a mixer for homogenization, and then approximately 2.5 kg of subsample was taken for chemical analysis using a quartering procedure.

### 2.3. Chemical Analyses

The whole body carcass samples were analyzed in duplicate for dry matter, ash, CP and crude fat according to the methods of AOAC [17]. The CP content was estimated by multiplying the total nitrogen content determined using a Kjeltec 8400 analyzer (FOSS Analytical AB, höganäs, Sweden) by 6.25. Crude fat was determined using an automatic extractor analyzer (XT 15i, Ankom Technology Co., Macedon, NY, USA).

### 2.4. Serum Biochemical Parameters

Serum total protein, albumin, urea, glucose, triglyceride and creatinine were determined using a Selectra Pro XL automatic biochemical analyzer (Vital Scientific, Dieren, The Netherlands) and commercial kits (BioSino Bio-Technology & Science Inc., Beijing, China).

### 2.5. DNA Extraction and 16S rRNA Amplification

Total genome DNA from each sample of colonic digesta was extracted using the QIAamp PowerFecal DNA Kit (Qiagen, Hilden, Germany) according to the manufacturer’s protocol. The DNA concentration of each sample was quantified using a Nanodrop 2000 spectrophotometer (Thermo Fisher Scientific, Waltham, MA, USA). The V3-V4 region of all bacterial 16S rRNA genes were amplified with the universal forward primer 338F (50-ACTCCTRCGGGAGGCAGCAG-30) and the reverse primer 806R (50-GGACTACCVGGGTATCTAAT-30). Polymerase chain reaction (PCR) amplicons were purified using the Qiagen Gel Extraction Kit (Qiagen, Hilden, Germany) according to the manufacturer’s recommendations. Sequencing libraries were generated using TruSeq^®^ DNA PCR-Free Sample Preparation Kit (Illumina, San Diego, CA, USA) following manufacturer’s recommendations and index codes were added. The library quality was then assessed on the Qubit@ 2.0 Fluorometer and Agilent Bioanalyzer 2100 system. Finally, the library was sequenced on an Illumina NovaSeq platform and 250 bp paired-end reads were generated. Bioinformatics analysis was performed as described previously [18].

### 2.6. Microbial Metabolite Analysis

The concentrations of short-chain fatty acids (SCFAs) from each sample in the colon were determined by gas chromatograph (7890B-7000D, Agilent Technologies, Santa Clara, CA, USA) according to the method described in a previous study [19]. Briefly, 0.1 g of colonic digesta was mixed with 2 mL of 25% metaphosphoric acid solution and vortexed to homogenize. The mixture was extracted with 2 mL diethyl ether for 10 min and then centrifuged at 4000× *g* for 10 min at 4 °C. The supernatant was put through a 0.22 μm filter and then injected for analysis. Helium was used as the carrier gas with a flow rate of 1mL/min without a spilt and the column HP-INNOWAX (25 m × 0.20 mm × 0.40 μm) was used to separate the composition. The column temperature was programmed as followed: the initial temperature was 100 °C held for 5min, then increased to 150 °C at 5 °C/min, and finally increased to 240 °C at 30 °C/min and held for 30 min. The transfer line and ion source temperature were kept at 250 °C and 200 °C, respectively. The detector was operated in an electron impact ionization mode (electron energy 70 eV) using a full scan and a single ion monitoring mode. In addition, an external standard calibration curve was used to determine the concentration of SCFAs in each sample.

To analyze the metabolite profiles, 0.1 g of colonic digesta was grounded with liquid nitrogen and the homogenate was resuspended with prechilled 80% methanol and 0.1% formic acid by well vortexing. The homogenate was incubated on ice for 5 min and then centrifuged at 15,000 rpm for 5 min at 4 °C. The supernatant was diluted with LC-MS grade water to a final concentration containing 53% methanol and was then centrifuged at 15,000× *g* for 10 min at 4 °C. Finally, the supernatant was injected into the LC-MS/MS system analysis. LC-MS/MS analyses were performed using a Vanquish UHPLC system (Thermo Fisher) coupled with an Orbitrap Q Exactive series mass spectrometer (Thermo Fisher). Samples were injected onto an Hypersil Gold column (100 × 2.1 mm^2^, 1.9 μm) using a 16-min linear gradient at a flow rate of 0.2 mL/min. The eluents for the positive polarity mode were eluent A (0.1% formic acid) and eluent B (methanol). The eluents for the negative polarity mode were eluent A (5 mM ammonium acetate, pH 9.0) and eluent B (methanol). The solvent gradient was set as follows: 2% B, 1.5 min; 2–100% B, 12.0 min; 100% B, 14.0 min; 100–2% B, 14.1 min; 2% B, 17 min. A Q Exactive series mass spectrometer was operated in positive/negative polarity mode with spray voltage of 3.2 kV, capillary temperature of 320 °C, sheath gas flow rate of 35 arb and aux gas flow rate of 10 arb. The raw UHPLC-MS/MS data files were processed using the Compound Discoverer 3.1 (CD3.1, Thermo Fisher Scientific Waltham, MA, USA) to perform peak alignment, peak picking, and quantitation for each metabolite. The main parameters were set as follows: retention time tolerance, 0.2 min; actual mass tolerance, 5 ppm; signal intensity tolerance, 30%; signal/noise ratio, 3; and minimum intensity, 100,000. After normalization to the total spectral intensity, the discriminated metabolites were selected based on variable importance in the projection (VIP) value from the partial least squares discriminant analysis (PLS-DA) using metaX. Metabolites with VIP > 1 and *p* < 0.05 and fold change (FC) ≥ 1.20 or ≤ 0.83 were considered statistically significant.

### 2.7. Statistical Analysis

Statistical analyses were performed using SAS 9.4 (SAS Institute Inc., Cary, NC, USA). Before comparing the differences between the groups, the normal distribution of the variables was assessed with the Shapiro–Wilk test. The variables accorded with normal distribution were analyzed by Student’s *t*-test, while those accorded with non-normal distribution were analyzed by Wilcoxon test. Statistical significance was declared at *p* < 0.05 and tendencies declared at 0.05 < *p* < 0.10. Data are presented as means ± SEM.

## 3. Results

### 3.1. Growth Performance

The ADG and G:F during the restriction phase as well as BW at day 14 were significantly decreased (*p* < 0.05) in the LP group compared with the NP group (Table 2). However, no significant differences (*p* > 0.05) were observed between the two groups for ADG and ADFI and G:F during the realimentation phase and the overall experiment. Besides, there was no difference (*p* > 0.05) in the experimental days to reach target BW between the two groups. Piglets in the LP group tended to have lower (*p* < 0.10) diarrhea incidence during the restriction phase and the whole experiment period.

### 3.2. Body Composition

Compared with the NP group, the LP group showed a significantly decreased body protein content (*p* < 0.05) at day 14, but not at the end of the experiment (Table 3). Piglets in the LP group showed a higher body lipid content both at day 14 and the end of the experiment than those in the NP group, but there was no statistical significance (*p* > 0.05). No differences (*p* > 0.05) were observed in the body water and ash content of piglets between the two groups at day 14 and the end of the experiment.

### 3.3. Serum Biochemical Parameters

At day 14, piglets in the LP group had lower (*p* < 0.05) concentrations of serum albumin and urea, and tended to have lower (*p* < 0.10) serum total protein concentration than those in the NP group (Table 4). At the end of the experiment, no differences (*p* > 0.05) were detected in serum biochemical parameters between the two groups.

### 3.4. Colonic Bacterial Community Structure

Compared with the NP group, the LP group had higher observed species, ACE and Chao1 indexes, which were not statistically significant at day 14 (*p* > 0.05), but were statistically significant (*p* < 0.05) at the end of the experiment (Table 5).

**Figure 1:**
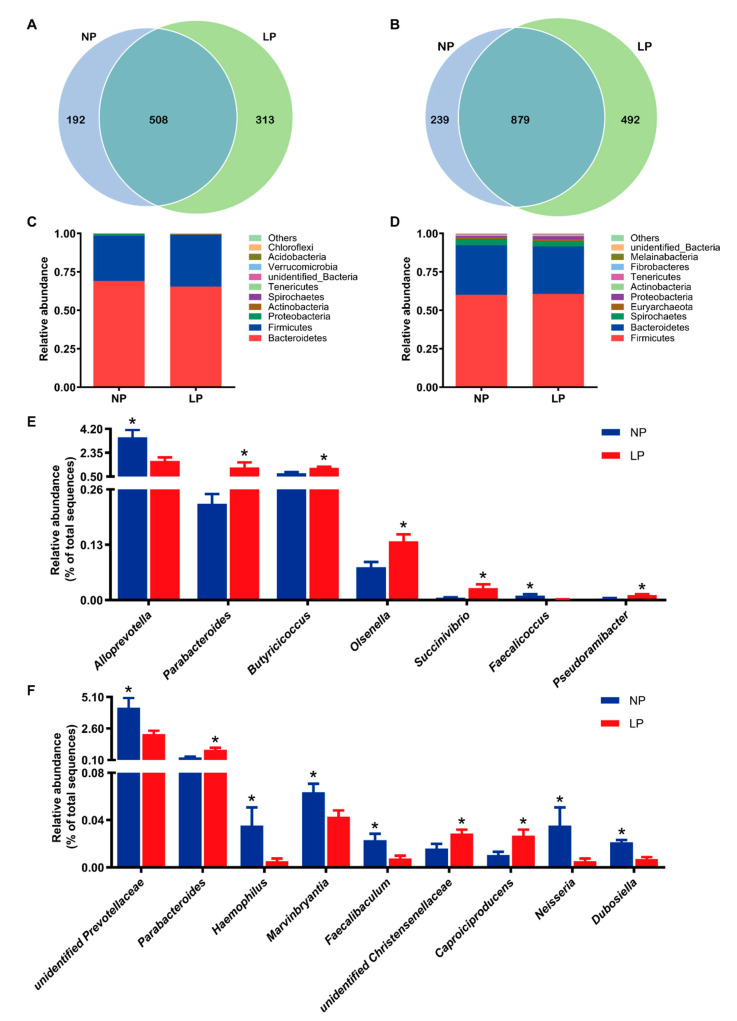
Effects of protein restriction and realimentation on the gut microbiota in the colonic digesta of piglets. Venn diagram showing the unique and shared OTUs between groups at day 14 (**A**) and the end of the experiment (**B**). Stacked bar chart depicting the relative abundance of top 10 phylum between groups at day 14 (**C**) and the end of the experiment (**D**). Bar chart showing the significantly changed bacteria genera between groups at day 14 (**E**) and the end of the experiment (**F**). * *p*-value < 0.05. NP, normal protein group. LP, low protein group.

At day 14, there were 192 and 313 unique operational taxonomic units (OUTs) in the NP and LP group, respectively, and a total of 508 shared OTUs in the two groups (Figure 1A). At the end of the experiment, 239 and 492 unique OTUs were identified in the NP and LP group, respectively, and 879 shared OTUs between the groups (Figure 1B). The composition of top 10 phylum at day 14 and the end of the experiment were shown in Figure 1C,D, respectively. Bacteroidetes and Firmicutes were the two dominant bacteria, accounting for 69.1 and 29.2% in the NP group, and 65.4 and 33.7% in the LP group at day 14, respectively, and accounting for 32.3 and 60.0% in the NP group, and 30.9 and 60.6% in the LP group at the end of the experiment, respectively. Compared with the NP group, increases in the ratio of Firmicutes to Bacteroidetes were observed in the LP group both at day 14 (0.52 and 0.43, respectively) and the end of the experiment (2.02 and 1.87, respectively), but was not statistically significant (*p* > 0.05). At the genus level, compared with the NP group, the relative abundance of *Parabacteroides*, *Butyricicoccus*, *Olsenella*, *Succinivibrio* and *Pseudoramibacter* were significantly increased (*p* < 0.05) in the LP group, and the relative abundance of *Alloprevotella* and *Faecalicoccus* were significantly decreased (*p* < 0.05) at day 14 (Figure 1E). At the end of the experiment, the piglets in the LP group showed a higher (*p* < 0.05) colonic relative abundances of *Parabacteroides*, unidentified *Christensenellaceae* and *Caproiciproducens*, and a lower (*p* < 0.05) relative abundance of unidentified *Prevotellaceae*, *Haemophilus*, *Marvinbryantia*, *Faecalibaculum*, *Neisseria* and *Dubosiella* than those in the NP group (Figure 1F).

### 3.5. Metabolite Profiles in the Colonic Digesta

**Figure 2:**
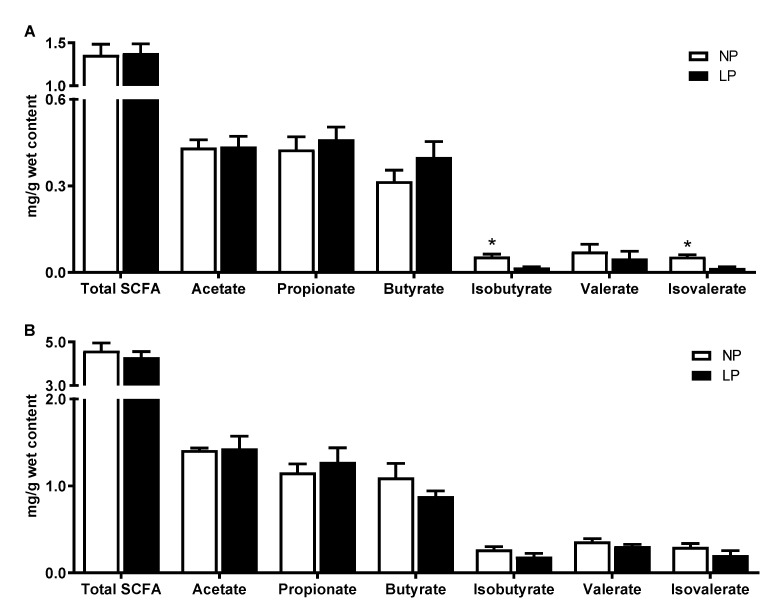
Effects of protein restriction and realimentation on SCFAs in colonic digesta of piglets at day 14 (**A**) and the end of the experiment (**B**). NP, normal protein group. LP, low protein group. * *p*-value < 0.05.

For SCFAs, at day 14, piglets in the LP group had lower (*p* < 0.05) isobutyrate and isovalerate concentrations compared with the NP group (Figure 2A). At the end of the experiment, the concentrations of SCFAs in colonic digesta did not show a significant statistical difference between the two groups (*p* > 0.05; Figure 2B).

**Figure 3:**
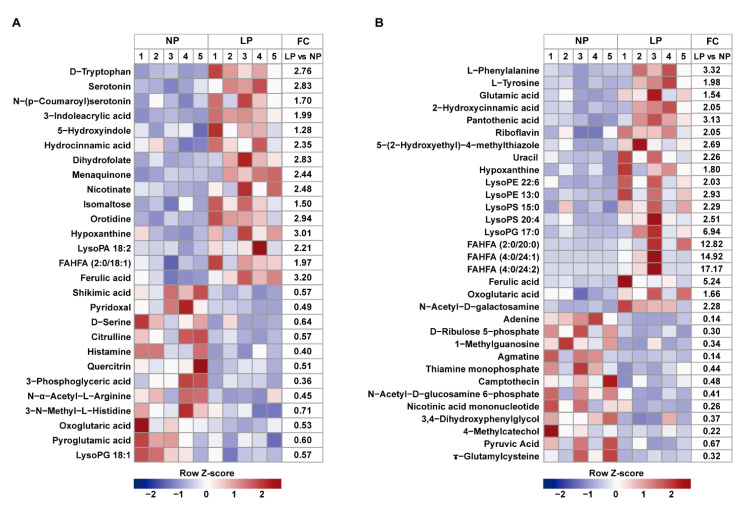
Effects of protein restriction and realimentation on colon metabolic profiles of piglets. Heatmap for the differentiated metabolites with VIP >1 and *p* < 0.05 and fold change (FC) ≥ 1.20 or ≤0.83 at day 14 (**A**) and the end of the experiment (**B**). Metabolites peak area were Z score transformed. Each sample is visualized in a single column and each metabolite is represented by a single row. The relative metabolite level is depicted according to the color scale. Red color indicates upregulation, and blue color indicates downregulation. NP, normal protein group. LP, low protein group.

Other metabolites in colonic digesta were further analyzed by LC-MS. At day 14 and the end of the experiment, 27 and 32 metabolites were identified with PLS-DA model VIP > 1 and *p* < 0.05 and fold change (FC) ≥ 1.20 or ≤0.83, respectively. These differentiated metabolites mainly included amino acids, lipids, carbohydrates, nucleotides, and others (Figure 3A,B). Compared with the NP group, there were 15 up-regulated metabolites and 12 down-regulated metabolites at day 14, and 20 up-regulated metabolites and 12 down-regulated metabolites at the end of the experiment in the LP group, respectively. Further metabolic pathways showed that during the protein restriction phase, the compounds involved in tryptophan metabolism, such as D-tryptophan, serotonin and 3-indoleacrylic acid, and the compounds involved in vitamin metabolism, such as dihydrofolate, menaquinone and nicotinate, were significantly increased, and the compounds involved in histidine metabolism, such as histamine and 3-N-methyl-L-histidine were significantly decreased in the LP group. During the protein realimentation phase, glycerophospholipid metabolism was mainly affected by LP diet, which was characterized by an increase in the concentrations of lysophosphatidylethanolamine (LysoPE 13:0 and LysoPE 22:6), lysophosphatidylserine (LysoPS 15:0 and LysoPS 20:4) and lysophosphatidylglycerol (LysoPG 17:0) compared with the NP diet. Fatty acid esters of hydroxy fatty acids (FAHFAs) metabolism was also altered, as reflected by the increased levels of FAHFA (2:0/20:0), FAHFA (4:0/24:1) and FAHFA (4:0/24:2) in the LP group.

### 3.6. Correlation between Microbiota Community and Metabolites

**Figure 4:**
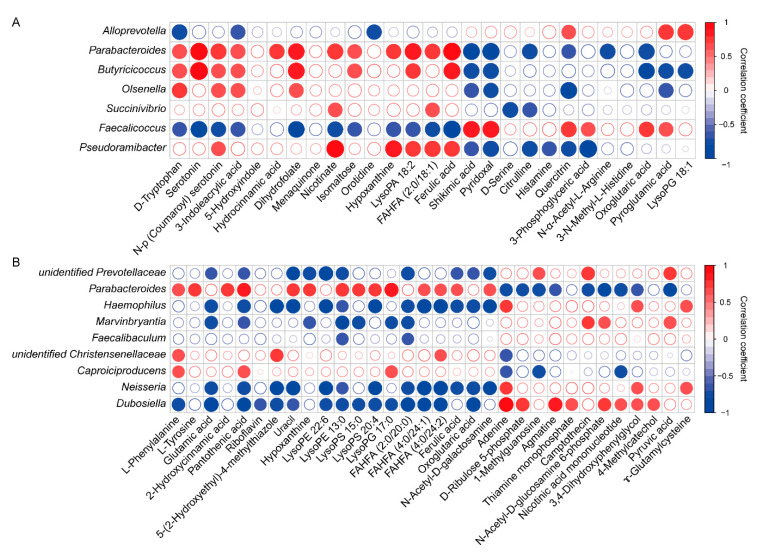
Correlation between the colonic microbiota (at the genera level) and metabolites during the protein restriction (**A**) and realimentation phase (**B**). The circle border color is based on the Spearman correlation coefficient distribution, and its size is based on the correlation coefficient value. The red-filled circle represents a significant positive correlation (*p* < 0.05), the blue-filled circle represents a significant negative correlation (*p* < 0.05) and the white-filled circle represents no significant correlation (*p* > 0.05).

A Spearman’s correlation analysis was used to explore the correlations between the colonic microbiota and their metabolite profiles (Figure 4A,B). The results showed that during the protein restriction phase, the relative abundances of *Parabacteroides*, *Butyricicoccus* and *Olsenella* were positively correlated with D-tryptophan, N-(p-coumaroyl) serotonin, 3-indoleacrylic acid and dihydrofolate, while they were negatively correlated with shikimic acid and pyridoxal. The relative abundances of *Alloprevotella* and *Faecalicoccus* had a negative correlation with D-tryptophan and 3-indoleacrylic acid, but a positive correlation with quercitrin and pyroglutamic acid. The concentrations of citrulline and histamine showed a negative correlation with the relative abundance of *Pseudoramibacter*. During the protein realimentation phase, the relative abundances of unidentified *Prevotellaceae* and *Marvinbryantia* were positively correlated with camptothecin and pyruvic acid, while they were negatively correlated with glutamic acid, pantothenic acid, hypoxanthine, LysoPE 13:0 and FAHFA (2:0/20:0). The relative abundances of *Haemophilus*, *Neisseria* and *Dubosiella* were positively correlated with adenine, 3,4-dihydroxyphenylglycol, while they were negatively correlated with glutamic acid, pantothenic acid, 5-(2-hydroxyethyl)-4-methylthiazole, uracil, LysoPE 22:6, LysoPE 13:0, LysoPS 20:4, FAHFA (2:0/20:0), FAHFA (4:0/24:1), FAHFA (4:0/24:2) and oxoglutaric acid. The relative abundances of *Parabacteroides* and *Caproiciproducens* were positively correlated with L-phenylalanine, pantothenic acid and LysoPG 17:0, while they were negatively correlated with adenine, 1-methylguanosine and nicotinic acid mononucleotide.

## 4. Discussion

It is generally assumed that protein restriction decreases the risk of post-weaning diarrhea, which may be due to the decrease of microbial fermentation of undigested protein in the gastrointestinal tract [20]. Kil and Stein [21] suggested that dietary CP levels in piglets should be reduced to below 18% during the immediate post-weaning period when in-feed antibiotic was not used. However, a deficient dietary CP supply can result in a reduction in piglet performance because CP supply is essential for animal growth and body protein deposition. Research has shown that protein restriction and subsequent realimentation can reduce diarrhea incidence in weaned piglets without negatively affecting their ultimate growth performance [6]. Similarly, in the present study, piglets in the LP group tended to have lower diarrhea incidence during the restriction phase and the whole experiment period. Compared with the NP group, the decreased growth performance of piglets in the LP group was observed during the restriction phase, but not during the protein realimentation phase and the overall experiment. Additionally, there was no difference in the experimental days to reach target BW between the LP and NP groups. Collectively, these results indicated that piglets in the LP group had a moderate ability for compensatory growth. It should be noted that the ability for compensatory gain depends upon the extent, timing and duration of nutrient restriction [22,23]. Piglets in the LP group had a lower body protein content in the protein restriction phase, which was consistent with other studies [24,25]. A reduction in dietary CP content suppressed protein synthesis in the liver, pancreas, kidney, and longissimus muscle of piglets, which may be due to decreased AA availability and inhibition of mammalian target of rapamycin signaling pathway [26]. However, no differences were observed in the chemical body composition at the end of the experiment between the LP and NP groups, indicating that the compensatory protein growth occurred. Similarly, previous studies found that nutritional history during the growing period had a compensatory effect on subsequent protein deposition during the finishing period [27,28].

Serum biochemical parameters reflect the body metabolism of piglets. An increase in serum total protein and albumin concentration was considered to be related to the age and rapid growth of piglets [29]. Serum urea can be used as an indicator to reflect the protein quality of diet and the nitrogen intake of animals and a response parameter to determine the protein requirement of animals [30]. Serum triglyceride concentration is a useful criterion for evaluating lipometabolic status [31]. Creatinine is the catabolite of creatine phosphate in muscles and can be used as a measure of muscle turnover [32]. In the present study, piglets in the LP group had lower urea concentrations that exhibited higher nitrogen utilization efficiency, which was consistent with previous studies [33,34]. However, there was no difference when the dietary CP level was adjusted to normal during the realimentation phase.

Gut microbiota and its fermentation metabolites play an important role in human and animal health. Diet is one of the main factors that affect the intestinal bacterial community [35]. In the present study, Firmicutes and Bacteroidetes constituted the two predominant phyla both at day 14 and the end of the experiment, which was consistent with another study [36]. In addition, age-related changes in the relative abundance of Firmicutes and Bacteroides were observed. As the animals aged, the relative abundance of Firmicutes increased, while the relative abundance of Bacteroidetes decreased. These changes in microbiome development were in agreement with other studies [37,38]. Evidence suggests that the increased ratio of Firmicutes to Bacteroidetes is an indicator of promoting lipid accumulation [39]. Compared with the NP group, increases in the ratio of Firmicutes to Bacteroidetes were observed in the LP group both at day 14 and the end of the experiment, which was consistent with the higher body lipid content in the LP group in these two phases, although the differences in these variables did not reach a significant level. In this study, we found that LP diet increased the relative abundance of several SCFA-producing bacteria including *Parabacteroides*, *Butyricicoccus*, *Olsenella* and *Pseudoramibacter* during the restriction phase, which can stimulate intestinal epithelial cells growth and protect the gut barrier [40,41,42]. The relative abundance of *Succinivibrio* was also significantly increased in the LP group, and *Succinivibrio* might be involved in starch, hemicellulose, and xylan degradation [43]. Meanwhile, piglets in the LP group had lower abundances of *Alloprevotella* and *Faecalicoccus*, which were considered as harmful bacteria. Previous studies showed that an increase in the abundance of *Alloprevotella* was associated with weight loss and inflammation [44,45,46]. *Faecalicoccus* was identified as an important feature to classify Crohn’s disease (a chronic and progressive inflammatory bowel disease) and healthy subjects, and its abundance in disease cohorts was significantly increased [47]. During the realimentation phase, the piglets in the LP group had higher abundances of beneficial bacteria such as *Parabacteroides*, unidentified *Christensenellaceae* and *Caproiciproducens*; however, they had lower harmful bacteria such as unidentified *Prevotellaceae*, *Marvinbryantia*, *Neisseria* and *Dubosiella* than those in the NP group. For example, *Christensenellaceae* is emerging as an important player in human health due to its role in protein and fiber fermentation [48]. *Caproiciproducens* is known as caproic acid-producing bacterium and its products can inhibit pathogenic bacteria and enhance animal immunity [49]. Evidence has shown that low fermentation of nitrogenous compounds can lead to a low abundance of *Prevotellaceae* [50]. *Marvinbryantia* is a pro-inflammatory bacterium with a negative correlation with antioxidant capacity [51,52]. *Dorea* is linked with gut inflammation, such as Crohn’s disease [53,54]. *Neisseria*, a Gram-negative aerobic cocci, can produce acids from diverse sugars and some of its species are pathogenic [55]. *Dubosiella* was associated with obesity-related microbial dysbiosis and was negatively associated with fecal SCFAs [56]. Collectively, these findings suggested that protein restriction and subsequent realimentation can lead to a healthier pattern of colonic bacterial community, which may be beneficial to the gut health of weaned piglets.

SCFA, mainly composed of acetate, propionate and butyrate, are the main products of carbohydrate fermentation by bacteria in the gut. SCFA not only has a trophic effect on intestinal epithelium, but also creates a slightly acidic environment to prevent the growth of acid-sensitive harmful bacteria such as pathogenic *Salmonella* and *Escherichia coli* [57]. As mentioned earlier, during the restriction phase, LP diet increased the relative abundance of several SCFA-producing bacteria such as *Parabacteroides*, *Butyricicoccus* and *Olsenella*, resulting in an increase in the concentrations of SCFA, but did not reach a significant level. Isobutyrate and isovalerate, as branched-chain fatty acids, originate exclusively from valine and leucine fermentation by gut microbiota, respectively, and can serve as a good marker of protein breakdown in the gut [58]. Piglets in the LP group had lower concentrations of isobutyrate and isovalerate in the colonic content during the restriction phase than those in the NP group, indicating that LP diet decreased the microbial fermentation of undigested protein in the gastrointestinal tract. Further metabolomics showed that LP diet mainly increased the compounds involved in tryptophan metabolism, such as D-tryptophan, serotonin, N-(p-Coumaroyl)serotonin, 3-indoleacrylic acid and 5-hydroxyindole in the restriction phase. These compounds are mainly produced by gut microbiota through the action of tryptophan hydroxylases and decarboxylases, or tryptophanases, and are beneficial to the regulation of host immune and metabolic homeostasis [59]. For example, D-tryptophan has been identified as an immunomodulatory probiotic substance that mediates Th2 cells to produce cytokines and chemokines, and promotes the proliferation of intestinal Treg cells [60]. Serotonin, an important gastrointestinal signaling molecule in the intestine, plays a beneficial role in establishing a healthy microenvironment in the intestine through modulation of cytokine levels [61]. Additionally, most of the indole derivatives are aryl hydrocarbon receptor (AhR) agonists, and AhR activation is essential for modulating metabolism and immune function [62]. The decreased concentrations of citrulline and histamine in the present study suggested that LP diet could prevent inflammatory response in the gastrointestinal tract [63,64]. During the protein realimentation phase, piglets in the LP group had increased concentrations of phenylalanine, tyrosine and glutamic acid. They are important precursors of neurotransmitters and signal transduction proteins and could participate in epithelial crosstalk and gut microbiota-mediated regulation of the host central nervous system [65]. A previous study indicated that one of the reasons for the weaning stress-induced gut microbiota dysbiosis might be due to the suppression of the phenylalanine metabolism, which then affected the piglet health through the microbiota–brain–gut axis [66]. It was also found that the compounds involved in glycerophospholipid metabolism and FAHFA metabolism were up-regulated in the LP group during the realimentation phase. Glycerophospholipids could strengthen the intestinal barrier and may have significant health implications at both enteric and somatic level [67,68,69]. FAHFAs, which are a novel class of endogenous bioactive lipids with health benefits and were first identified in mammalian white adipose tissue in 2014, are formed by esterification between the hydroxyl group of a hydroxy fatty acid and the carboxyl group of a fatty acid [70]. Among FAHFAs, the family of palmitic acid esters of hydroxy stearic acids (PAHSAs) are well studied, which have a wide range of anti-inflammatory and anti-diabetic effects [70,71,72]. In addition to PAHSAs, only a limited number of studies have focused on other FAHFAs [73]. Several previous studies have shown that polyunsaturated FAHFAs exert immunomodulatory effects [74,75]. Recently, several new short chain FAHFAs (SFAHFAs) of acetic acid or propanoic acid esterified long chain hydroxy fatty acids were identified in the large intestine of mice and levels of SFAHFAs tended to be decreased in mice fed with a high-fat diet compared with those fed with a normal diet [76]. Intestinal microbiota has been proposed to be an important endogenous source of vitamins, especially of the K and B groups [77]. In the present study, piglets in the LP group had higher concentrations of menadione, dihydrofolic acid and nicotinic acid at day 14, and pantothenic acid, riboflavin and 4-methyl-5-thiazoleethanol at the end of the experiment. From the host perspective, these compounds can act as vitamins with nutritional/physiological properties. In addition, they are known to play an important role in regulating immune homeostasis, which has been discussed in detail elsewhere [78,79,80]. Overall, these results indicated that the LP diet may have more metabolites that play a role in animal growth and gut health during the protein restriction and the subsequent realimentation phase.

## 5. Conclusions

The present study demonstrated that protein restriction and subsequent realimentation lead to compensatory growth and compensatory protein deposition in piglets, and contribute to animal intestinal health by altering the gut microbiota and metabolite profiles. These findings may provide new insights into the application of nutritional strategy to maintain animal and human gut health.

## Figures and Tables

**Table 1:** Ingredient and nutrient composition of experimental diets in piglets (% as-fed basis).

Item	Restriction Phase d 0 to 14		Realimentation Phase d 15 to 25 kg of BW
	NP-20%	LP-16%	NP-19%
Ingredients, %			
Corn	34.94	45.08	68.43
Expanded corn	10.00	10.00	
Expanded soybean	8.00	4.00	
Soybean meal, enzyme treated	10.00	7.92	10.00
Soybean meal	3.20		14.46
Fish meal	3.20	2.00	2.00
Whey protein concentrate	3.20	2.00	
Whey powder	15.00	15.00	
Yeast extract	2.00	2.00	
Soybean hulls	2.00	2.00	
Soybean oil	1.00	1.00	1.00
Sucrose	2.50	2.50	
Salt	0.20	0.20	0.35
Dicalcium phosphate	1.05	1.47	0.55
Limestone			0.93
Calcium Citrate	0.90	0.88	
L-Lys HCl	0.59	0.99	0.47
DL-Met	0.10	0.18	0.06
L-Thr	0.19	0.37	0.12
L-Trp	0.02	0.09	0.01
L-Val	0.11	0.32	
L-Ile		0.20	
Phytase	0.02	0.02	0.02
Zinc oxide	0.18	0.18	
Choline chloride	0.20	0.20	0.20
Titanium dioxide	0.40	0.40	0.40
Premix ^1^	1.00	1.00	1.00
Total	100.00	100.00	100.00
Calculated nutrient composition ^2^, %			
NE ^3^, MJ/kg	10.82	10.92	10.50
SID ^4^ Lys	1.41	1.41	1.23
SID ^4^ Met	0.41	0.41	0.36
SID ^4^ Thr	0.83	0.83	0.73
SID ^4^ Trp	0.23	0.23	0.20
SID ^4^ Val	0.89	0.89	0.78
SID ^4^ Ile	0.73	0.72	0.71
Calcium	0.82	0.82	0.75
Total phosphorus	0.72	0.72	0.61
Analyzed nutrient composition, %			
CP ^5^	20.09	16.33	19.35

^1^ Provided, per kilogram of diet, 12 400 IU vitamin A, 2 800 IU vitamin D3, 30 mg vitamin E, 5 mg vitamin K3, 3 mg thiamin, 10 mg riboflavin, 40 mg niacin, 8 mg pyridoxine, 40 μg vitamin B12, 0.08 mg biotin, 15 mg pantothenic acid, 1 mg folic acid, 80 mg Zn, 120 mg Fe, 70 mg Mn, 16 mg Cu, 0.7 mg I, 0.48 mg Se. ^2^ Values were calculated according to NRC [15]. ^3^ NE, net energy. ^4^ SID, standardized ileal digestible. ^5^ CP, crude protein.

**Table 2:** Effects of protein restriction and realimentation on the growth performance of piglets ^1^.

Item	NP	LP	SEM	*p*-Value
Restriction phase (day 0 to 14)				
Initial BW, kg	6.39	6.38	0.02	0.861
ADG, g	324	261	18.1	0.041
ADFI, g	418	372	17.0	0.093
G:F	0.77	0.70	0.02	0.022
Diarrhea incidence, %	2.00	0.29	0.55	0.060
BW at end of phase, kg	10.9	10.0	0.26	0.043
Realimentation phase (day 15 to target BW)				
ADG, g	524	543	7.9	0.153
ADFI, g	859	888	10.1	0.086
G:F	0.61	0.61	0.01	1.000
Diarrhea incidence, %	5.33	2.61	1.21	0.151
Final BW, kg	24.9	25.1	0.23	0.579
Overall (day 0 to target BW)				
ADG, g	455	452	9.5	0.814
ADFI, g	708	718	8.2	0.413
G:F	0.64	0.63	0.01	0.283
Diarrhea incidence, %	3.76	1.56	0.82	0.095
Days on experiment	40.8	41.5	0.81	0.537

^1^ BW, body weight. ADG, average daily gain. ADFI, average daily feed intake. G:F, gain:feed. NP, normal protein group. LP, low protein group.

**Table 3:** Effects of protein restriction and realimentation on the chemical body composition of piglets ^1^.

Item	NP	LP	SEM	*p*-Value
Day 14 of the experiment				
Water, g/kg	709	708	4.3	0.845
Protein, g/kg	163	157	1.8	0.034
Lipid, g/kg	90.1	97.3	4.24	0.261
Ash, g/kg	31.0	31.4	1.40	0.846
End of the experiment				
Water, g/kg	694	689	4.6	0.427
Protein, g/kg	168	166	1.6	0.451
Lipid, g/kg	107	113	6.5	0.523
Ash, g/kg	28.2	27.1	1.46	0.622

^1^ NP, normal protein group. LP, low protein group.

**Table 4:** Effects of protein restriction and realimentation on the serum biochemical parameters of piglets ^1^.

Item	NP	LP	SEM	*p*-Value
Day 14 of the experiment				
Total protein, g/L	51.2	46.1	1.79	0.085
Albumin, g/L	29.6	23.8	1.31	0.017
Urea, mmol/L	2.82	1.66	0.25	0.018
Glucose, mmol/L	7.57	6.48	0.55	0.203
Triglyceride, mmol/L	0.49	0.50	0.05	0.845
Creatinine, μmol/L	71.3	61.8	5.30	0.245
End of the experiment				
Total protein, g/L	56.8	54.8	1.48	0.357
Albumin, g/L	31.8	29.0	1.08	0.104
Urea, mmol/L	3.97	4.50	0.58	0.543
Glucose, mmol/L	4.46	4.04	0.58	0.621
Triglyceride, mmol/L	0.48	0.49	0.04	0.908
Creatinine, μmol/L	83.2	83.9	5.33	0.919

^1^ NP, normal protein group. LP, low protein group.

**Table 5:** Effects of protein restriction and realimentation on the alpha-diversity of colonic microbiota of piglets ^1^.

Item	NP	LP	SEM	*p*-Value
Day 14 of the experiment				
Observed species	377	420	30.3	0.345
ACE	427	460	25.1	0.385
Chao1	503	591	37.0	0.143
Shannon	5.27	5.25	0.18	0.951
Simpson	0.92	0.92	0.01	0.872
End of the experiment				
Observed species	636	706	16.6	0.017
ACE	656	729	18.6	0.025
Chao1	643	714	17.6	0.022
Shannon	6.28	6.28	0.14	0.996
Simpson	0.96	0.96	0.01	0.681

^1^ NP, normal protein group. LP, low protein group.

## Data Availability

The data presented in this study are available on reasonable request from the corresponding author.
